# Albumin of People with Diabetes Mellitus Is More Reduced at Low HbA1c

**DOI:** 10.3390/ijms242216256

**Published:** 2023-11-13

**Authors:** Margret Paar, Gerhard Cvirn, Gerd Hoerl, Gilbert Reibnegger, Harald Sourij, Caren Sourij, Harald Kojzar, Karl Oettl

**Affiliations:** 1 Division of Medicinal Chemistry, Otto Loewi Research Center, Medical University of Graz, Neue Stiftingtalstrasse 6, 8010 Graz, Austria; margret.paar@medunigraz.at (M.P.); gerhard.cvirn@medunigraz.at (G.C.); gerd.hoerl@medunigraz.at (G.H.); gilbert.reibnegger@medunigraz.at (G.R.); 2 Division of Endocrinology and Diabetology, Interdisciplinary Metabolic Medicine Trials Unit, Medical University of Graz, Auenbruggerplatz 15, 8036 Graz, Austria; ha.sourij@medunigraz.at (H.S.); harald.kojzar@medunigraz.at (H.K.); 3 Division of Cardiology, Department of Internal Medicine, Medical University of Graz, Auenbruggerplatz 15, 8036 Graz, Austria; caren.sourij@medunigraz.at

**Keywords:** human serum albumin, human mercaptalbumin, human nonmercaptalbumin, diabetes mellitus, oxidative stress, biomarker

## Abstract

Oxidative stress is involved in the development, progression, and complications of diabetes mellitus (DM). Oxidative modification of human serum albumin’s cysteine-34 is a marker for oxidative stress-related pathological conditions. We aimed to evaluate the redox state of albumin in patients with DM to investigate possible correlations with age, diabetes duration, and disease control status. Plasma aliquots were collected from 52 participants (26 type 1 and 26 type 2 DM). Patients were divided into two groups according to their glycated hemoglobin levels less than or equal to and greater than 58 mmol/L. Albumin redox state was assessed with high-performance liquid chromatography by fractionating it into human mercaptalbumin (HMA) and human nonmercaptalbumin 1 and 2 (HNA1 and HNA2). Albumin redox fractions were differently related to the age of study participants. In age-matched T1DM and T2DM groups, the albumin redox state was essentially the same. Irreversibly oxidized HNA2 was positively correlated with diabetes duration, especially in the T1DM group. HNA was increased in people with an increased HbA1c (>58 mmol/mol). Our results support the hypothesis that oxidative stress plays a crucial role in DM pathogenesis and emphasize the importance of diabetes control on systemic oxidative burden.

## 1. Introduction

Diabetes mellitus (DM) summarizes a group of metabolic disorders having in common elevated blood glucose levels (hyperglycemia) and insufficient insulin production and/or action. DM is classified into two major types: type 1 (T1) DM, an autoimmune disorder that destroys pancreatic beta cells leading to insulin dependency, and type 2 (T2) DM, a complex metabolic disorder driven by insulin resistance (IR) and insufficient insulin production [1,2].

Despite different etiologies, patients of both DM types have an increased risk of micro- and macrovascular complications caused by several mechanisms, including hyperglycemia, hyperlipidemia, and oxidative stress [3]. The latter is characterized as an imbalanced equilibrium between pro- and antioxidants in favor of the prooxidants, which leads to a disruption of redox signaling and control and/or molecular damage [4]. This results in an excess of reactive species, such as reactive oxygen species (ROS), and causes degenerative modifications of proteins, lipids, and DNA.

Modified proteins often serve as established biomarkers for the diagnosis and control of several diseases, such as glycated hemoglobin A1c (HbA1c) in people with diabetes, reflecting the average glucose levels over the past few months [5]. In addition, by monitoring glucose levels over the past few weeks, the laboratory value of glycated albumin (GA), also denoted as glycated serum protein (GSP), can be used [6]. Although not routinely measured, albumin redox state has proven to be a biomarker for the estimation of systemic oxidative burden [7,8]. Given its high concentration, which is, e.g., about 20 times that of ascorbate and even higher than that of urate, and with its free thiol group at cysteine-34 (Cys-34), albumin may contribute a high proportion to the antioxidant capacity of human plasma [9]. Three fractions of albumin can be differentiated with regard to the redox state of Cys-34: (i) the reduced form named human mercaptalbumin (HMA), with Cys34 as a thiol; (ii) the mildly oxidized form, human nonmercaptalbumin 1 (HNA1), with Cys34 as a disulfide with a low molecular mass thiol; (iii) a strongly oxidized form, human nonmercaptalbumin 2 (HNA2), forming a sulfinic or sulfonic acid [10,11]. While the conversion of HMA to HNA1 is reversible, a conversion to HNA2 is supposed to be perpetual. In young, healthy individuals, the ratio of the three fractions is about 70–80% HMA to 20–30% HNA1 to 2–5% HNA2 [12,13]. During aging and various pathologies involving oxidative stress, such as DM, the albumin redox state is shifted towards more oxidized fractions, thereby reflecting disease status and even constituting prognostic power for disease progression and complications [14,15,16,17]. 

The likelihood of developing an oxidative stress-related disease increases with age. This makes it difficult to dissect the effects of age or disease on albumin redox state. We thus aimed to investigate possible differences of albumin redox fractions HMA, HNA1, and HNA2 between people with T1DM and T2DM and the association with patient age, diabetes duration, and glycaemic control.

## 2. Results

### 2.1. Albumin Redox State in Relation to Demographic and Laboratory Data of Diabetes Mellitus Patients

We evaluated the albumin redox state in the plasma of 52 participants with DM, 26 with type 1 and 26 with type 2 diabetes. Patient characteristics and albumin-related laboratory variables are given in Table 1.

HMA was decreased and HNA1 increased in T2DM patients compared to T1DM patients. Importantly, the two groups differed significantly in age (median 46.5 years in T1DM vs. 59.5 years in T2DM, *p* < 0.001) (Table 1). After age matching (median 56 vs. 57 years, *p* = 0.64) and considering only patients between 41–65 years, we could not observe any significant differences in the albumin redox state of T1DM and T2DM patients (Table 2).

Patient age affects all three albumin redox fractions, however, in a different manner in both groups. In T1DM, there was a negative correlation of age with HMA and a positive correlation with HNA1, whereas in T2DM patients, age was correlated with HNA2 only (Table 3).

### 2.2. Albumin Redox State in Relation to Diabetes Duration

Albumin redox state in the plasma of DM patients was associated with the disease duration. When analyzing the T1DM group solely, a negative correlation of HMA with the disease duration (*r* = −0.400, *p* = 0.048; corrected for age) and a positive correlation of HNA2 with the disease duration (*r* = 0.413, *p* = 0.04; corrected for age) were observed. No such correlations were observed when analyzing solely the T2DM group. In the combined patient group, a weak correlation of HNA2 with the disease duration was observed (*r* = 0.31, *p* = 0.03; corrected for age). Detailed data on correlations of albumin redox state with disease duration are given in Table 4.

### 2.3. Albumin Redox State in Relation to Disease Control Status

**Figure 1:**
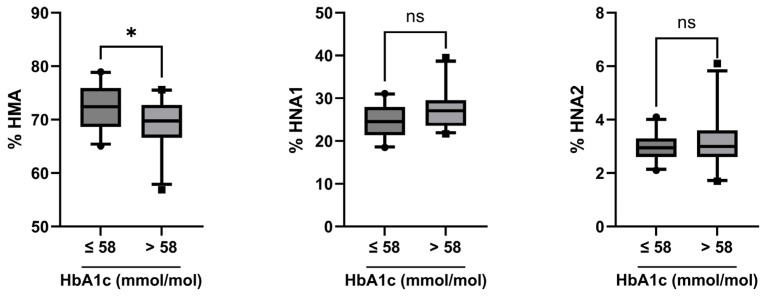
Albumin redox state in patients with HbA1c values ≤ or >58 mmol/mol. Albumin redox state was assessed in patients with HbA1c ≤ 58 mmol/mol (*n* = 28) and compared to patients with HbA1c > 58 mmol/mol (*n* = 24). Statistical significance was assessed by the Mann–Whitney U test. *, *p* ≤ 0.05; ns, not significant.

The combined group of all DM patients was divided into two groups based on their HbA1c values ≤ or >58 mmol/mol, Figure 1). The median HbA1c values were 52 vs. 65 mmol/mol in both DM groups. The glycated serum protein (GSP/Alb) content was increased, albeit marginally, in the group with higher HbA1c levels (0.6 mol GSP/mol albumin compared to 0.5 mol/mol, *p* = 0.02). There was no significant difference in the median age of these groups (median 53.5 vs. 57.0 years, *p* = 0.26). eGFR was higher in the group with lower HbA1c (median 99.3 vs. 86.1 mL/min/1.73 m^2^, *p* = 0.02). All available comorbidities were comparable between both groups (Table 5).

HMA fraction was somewhat higher in patients with HbA1c ≤ 58 mmol/mol (72.4 vs. 69.8%, *p* = 0.05) (Figure 1, left panel), and HNA fraction was decreased (median 27.6 vs. 30.3%, *p* = 0.05). Upon splitting HNA into its subfractions HNA1 and HNA2, there was a trend of lower HNA1 in patients with lower HbA1c (24.6 vs. 27.1%, *p* = 0.06), albeit not statistically significant, and no difference in HNA2 (3.0 vs. 3.0%, *p* = 0.61) (Figure 1, middle and right panel). 

## 3. Discussion

In the present study, we show, in good agreement with several other studies, that the albumin redox state of people with diabetes correlates with age, diabetes duration, and the quality of disease control. However, unlike existing studies, we did not look at albumin as a whole only but divided it into fractions with different degrees of oxidation. We found that the reversibly oxidized albumin fraction HNA1 increased with age in T1DM patients and that the irreversibly oxidized albumin fraction HNA2 increased with age in T2DM patients. The HNA2 fraction increased with diabetes duration in the T1DM group as well as in the combined T1DM + T2DM group. Most importantly, we found that the quality of disease control significantly affects the extent of albumin oxidation; plasma levels of the reduced albumin fraction HMA were significantly higher in patients with lower HbA1c than those with high HbA1c.

Several studies have reported increased Cys34 oxidation in DM patients compared to healthy subjects. These studies differ in the patient cohorts in terms of diabetes type, disease state, group size, or age [18,19,20,21]. Furthermore, different approaches have been applied to determine free thiol groups, total oxidized HNA fractions, or trioxidated Cys34 (corresponding to HNA2) in plasma [7,17,22,23]. However, studies reporting albumin redox state in terms of all three redox fractions HMA, HNA1, and HNA2 in parallel are scarce [14]. 

The percentage of HMA in all DM patients of the present study was within the range of healthy persons (63–73%) [12,13]. It is well known that the albumin redox state is shifted towards more oxidized fractions during aging, strenuous exercise, and several pathologies involving oxidative burden [15,18,20,24,25,26]. The effects of age or disease on the distribution of albumin redox fractions are sometimes difficult to dissect from each other. Also, in the present study, the decreased HMA and increased HNA1 fractions in T2DM patients compared to T1DM patients were strongly related to the different ages of the groups. After matching for age, no significant differences in the albumin redox state of T1DM and T2DM patients were left. Interestingly, the correlations of oxidized albumin fractions with age were different in the two groups. Whereas HNA1 was positively correlated with the age of T1DM patients, it was HNA2 in the T2DM group. Both groups cover a broad age range of 46 years in T1DM and 32 years in T2DM, respectively. T2DM patients were older in the median than T1DM patients. Based on previous reports, correlations of albumin redox state with age, as seen in the T1DM group, could be expected [25,27]. The difference in the T2DM group is interesting and may be attributed to redox processes related to the disease etiology. This observation merits further investigation, especially in the context of the consideration of oxidized albumin fractions as disease biomarkers.

A correlation between HNA and the disease duration in DM has been recently described by Kobayashi et al. [16]. Our results confirm these findings and further define that this correlation is mainly found in T1DM patients and is mainly driven by HNA2 fractions. 

In accordance with the findings of Suzuki et al. [20], HMA is somewhat decreased, and consequently, the total HNA fraction is increased in patients with high HbA1c compared to patients with lower HbA1c. The difference may appear small at first glance. However, one has to keep in mind that, given a concentration of 500 µmol/L of albumin in plasma, a decreased HMA fraction by 2.4% means a decrease of free thiol groups by 12 µmol/L. The increase in oxidized albumin in patients with high HbA1c is rather attributable to increased HNA1, although not significant, than the irreversibly oxidized HNA2 fraction. This may be therapeutically interesting as HNA1 constitutes a reversible modification and may be reduced by low-molecular-weight thiols such as N-acetyl-L-cysteine [28]. The importance of the redox situation in DM was emphasized by a recent paper reporting that not the glycation of albumin but the fraction of HNA1 was strongly associated with a decline in kidney function in DM patients [17].

Our study has some limitations. It was part of a large prospectively designed study, and we analyzed all samples available for albumin redox state determination; therefore, a power calculation was not performed. Thus, the T1DM and T2DM patient groups were small, and the group size may be insufficient to obtain conclusive results. It is necessary to confirm our findings in larger patient cohorts. The investigation of differences in albumin redox state between poorly and well-controlled DM patients within these groups in terms of a longitudinal prospective study would be especially interesting to investigate the impact of the different etiologies on the potential biomarkers HNA1 and HNA2.

In summary, our results confirm the view that in diabetes mellitus, systemic redox balance determined by albumin redox state is shifted towards oxidation. However, this may be improved by good glycemic control, which underlines the importance of the latter to keep oxidative burden at lower levels. Special attention should be paid to the patient’s age when considering albumin redox state as a disease biomarker. 

## 4. Materials and Methods

### 4.1. Study Patients

This study is part of the ‘Immune response to COVID-19 vaccination in people with Diabetes Mellitus—COVAC-DM’ study, described in detail previously (Sourij, Tripolt, Aziz, Diabetes Obes Metab 2022) and was approved by the ethics committee of the Medical University of Graz, Graz, Austria (ethical approval number: 33-366 ex 20/21). Briefly, adults aged 18–80 years with T1DM or T2DM, who were diagnosed with diabetes prior to receiving a COVID-19 vaccine and who were willing to give written informed consent, were included in the study. Venous blood was collected in pre-citrated Vacuette tubes containing 3.8% sodium citrate (Greiner Bio-one GmbH, Kremsmünster, Austria) and was centrifuged at 500 g for 20 min in order to prepare plasma samples. Of the original 161 individuals, plasma samples of 52 individuals prior to COVID-19 vaccination were available for the determination of the albumin redox state and are presented herein. Seven patients of the T2DM group presented with diagnosed liver disease; however, albumin redox state as well as albumin concentration was not altered in this subgroup compared to T2DM patients without liver disease (Table 6). Therefore, we did not exclude these patients from the analyses.

### 4.2. Determination of Albumin Redox State

**Figure 2:**
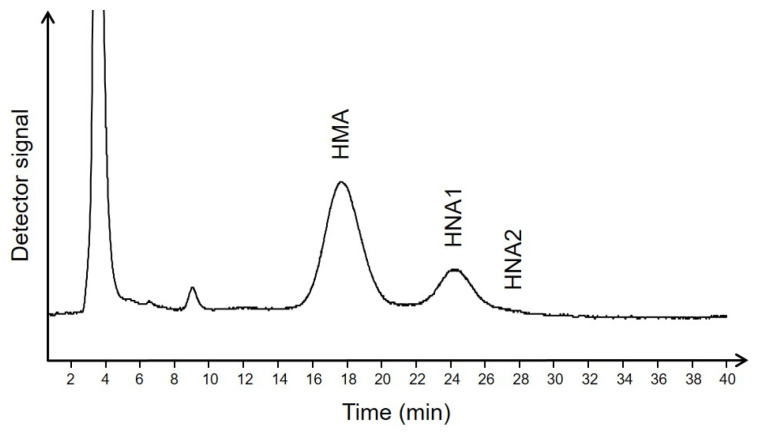
Exemplary HPLC chromatogram of albumin redox fractions in plasma of one patient with diabetes mellitus.

The redox state of albumin in terms of human mercaptalbumin (HMA), human nonmercaptalbumin 1 (HNA1), and human nonmercaptalbumin 2 (HNA2) was assessed by high-performance liquid chromatography (HPLC) according to the method of Hayashi et al. [29,30]. Briefly, sodium citrate plasma samples were diluted in a ratio of 1:100 in 0.1 M sodium phosphate, 0.3 M sodium chloride, pH 6.87, and filtered through a Whatman 0.45 µm nylon filter (Bartelt Labor-& Datentechnik, Graz, Austria). Twenty µL of each diluted sample were injected into an HPLC system using a Shodex Asahipak ES-502N 7 C anion exchange column (7.5 × 100 mm, Bartelt Labor-& Datentechnik, Graz, Austria) with 50 mM sodium acetate, 400 mM sodium sulfate, pH 4.85, as mobile phase (A). Gradient elution was achieved with the same mobile phase containing 10% ethanol (*v*/*v*, mobile phase B) at a flow rate of 1 mL/min. A linear gradient of 0 to 60% mobile phase (B) was used for elution. The column was kept at 35 °C. Detection was carried out by measuring fluorescence at 280/340 nm using a Jasco 821FP fluorescence detector (Spectronex, Strasnice, Czech Republic). Quantification was based on the individual peak areas as determined by PeakFit software (Version 4.12, SPSS Science, Chicago, IL, USA). A representative chromatogram is shown in Figure 2.

### 4.3. Determination of Albumin Related Diagnostic Laboratory Variables in Plasma

Albumin concentration was measured using the Albumin Bromocresol Green (BCG) Assay Kit (Abcam, Amsterdam, The Netherlands). Glycation of Albumin was assessed by Glycated Serum Protein Assay (Diazyme Europe GmbH, Dresden, Germany).

### 4.4. Statistics

Data were stored in a Microsoft Excel data sheet. Statistical analyses were performed using GraphPad Prism (Version 9; GraphPad Software, San Diego, CA, USA) and IBM SPSS Statistics (Version 27, Armonk, NY, USA). Results are presented as medians and 25th–75th percentile. Data distribution was assessed by the Kolmogorov-Smirnoff test. Differences between the groups were assessed by applying the Mann–Whitney U test (quantitative variables) or Fisher’s exact test (qualitative variables). Spearman (partial) correlations between variables were calculated. All *p*-values of ≤0.05 were considered statistically significant. * *p* ≤ 0.05, ** *p* ≤ 0.01, *** *p* ≤ 0.001

## Figures and Tables

**Table 1:** Demographic data, clinical data, and albumin-related laboratory variables of T1DM and T2DM patients.

	Combined Group *n* = 52	T1DM *n* = 26	T2DM *n* = 26	T1DM vs. T2DM *p* Value
*n* female (%)	19 (37)	10 (38)	9 (35)	>0.999
age (years)	56.0 (19–73)	46.5 (28.5–57.3)	59.5 (52.5–64.0)	**<0.001**
diabetes duration (years)	15.0 (2.0–51.0)	21.5 (10.5–40.8)	13.5 (7.8–19.5)	**0.03**
HbA1c (mmol/mol)	58 (50–64)	56 (45–61)	60 (56–66)	**0.04**
*n* with HbA1c > 58 mmol/mol (%)	24 (46%)	9 (35%)	15 (58%)	0.163
Albumin (g/dL)	3.9 (3.7–4.2)	3.9 (3.7–4.1)	3.9 (3.7–4.2)	0.65
GSP (µmol/L)	307 (256–398)	332 (281–464)	273 (233–377)	0.12
GSP/Alb (mol/mol)	0.6 (0.5–0.7)	0.6 (0.5–0.8)	0.5 (0.4–0.6)	0.14
% HMA	72.0 (68.3–74.8)	73.2 (70.8–76.2)	69.1 (66.7–72.4)	**<0.001**
% HNA1	25.6 (23.2–28.3)	23.4 (21.3–26.1)	27.3 (25.3–29.6)	**<0.001**
% HNA2	3.0 (2.6–3.4)	3.0 (2.5–3.5)	3.0 (2.6–3.4)	0.64

Data are presented as numbers (percent) or medians (25th–75th percentile). Differences between the groups were assessed by the Mann–Whitney U test or Fisher’s exact test. Significant differences are highlighted.

**Table 2:** Albumin redox state in plasma of age-matched T1DM and T2DM patients.

	T1DM *n* = 17	T2DM *n* = 23	*p* Value
% HMA	72.5 (68.7–75.2)	69.6 (68.1–72.4)	0.12
% HNA1	23.9 (22.3–27.9)	27.2 (24.8–29.2)	0.07
% HNA2	3.1 (2.5–3.7)	2.8 (2.6–3.3)	0.45

Data are presented as medians (25th–75th percentile). Differences between the groups were assessed by the Mann–Whitney U test.

**Table 3:** Non-parametric correlation coefficients of albumin redox fractions in plasma with the age of T1DM and T2DM patients.

Age				
		Combined Group	T1DM	T2DM
HMA	*r*	−0.57	−0.59	−0.29
	*p*	**<0.0001**	**0.002**	0.159
HNA1	*r*	0.53	0.55	0.17
	*p*	**<0.0001**	**0.004**	0.418
HNA2	*r*	0.37	0.34	0.46
	*p*	**0.007**	0.093	**0.017**

Significant correlations are highlighted.

**Table 4:** Non-parametric partial correlation coefficients of albumin redox fractions in plasma with the disease duration of T1DM and T2DM patients (control variable: patients’ age).

Disease Duration				
		Combined Group	T1DM	T2DM
HMA	*r*	−0.26	−0.40	−0.26
	*p*	0.066	**0.048**	0.206
HNA1	*r*	0.13	0.34	0.22
	*p*	0.376	0.102	0.281
HNA2	*r*	0.31	0.41	0.03
	*p*	**0.025**	**0.040**	0.894

Significant correlations are highlighted.

**Table 5:** Comorbidities, eGFR, and hemoglobin values of patients with HbA1c values ≤ or >58 mmol/mol.

	Combined Group *n* = 52	HbA1c ≤ 58 mmol/mol *n* = 28	HbA1c > 58 mmol/mol *n* = 24	HbA1c ≤ 58 vs. >58 mmol/mol *p* Value
*n* hypertension (%)	26 (50)	13 (46)	13 (54)	0.78
*n* coronary heart disease (%)	5 (10)	2 (7)	3 (13)	0.65
*n* myocardial infection (%)	1 (2)	1 (4)	0 (0)	>0.99
*n* TIA (%)	2 (4)	1 (4)	1 (4)	>0.99
*n* heart failure (%)	1 (2)	1 (4)	0 (0)	>0.99
*n* PTCA/CABG (%)	3 (6)	2 (7)	1 (4)	>0.99
*n* stroke (%)	2 (4)	1 (4)	1 (4)	>0.99
*n* liver disease (%)	7 (13)	3 (11)	4 (17)	0.69
*n* history of cancer (%)	4 (8)	2 (7)	2 (8)	>0.99
*n* retinopathy (%)	12 (23)	5 (18)	7 (29)	0.51
*n* polyneuropathy (%)	16 (31)	7 (25)	9 (38)	0.38
eGFR, mL/min/1.73m^2^	90.7 (80.2–102.0)	99.3 (84.6–111.3)	86.1 (68.3–94.1)	**0.02**
hemoglobin, g/dL	14.8 (14.0–16.0)	14.5 (13.6–15.6)	14.5 (13.7–15.9)	>0.99

Data are presented as numbers (percent) or medians (25th–75th percentile). Differences between the groups were assessed by the Mann–Whitney U test or Fisher’s exact test. Significant differences are highlighted. CABG, coronary artery bypass graft; eGFR, estimated glomerular filtration rate; PTCA, percutaneous transluminal coronary angioplasty; TIA, transient ischemic attack.

**Table 6:** Albumin concentration and albumin redox state in plasma of T2DM patients with and without liver disease.

	Liver Disease *n* = 7	No Liver Disease *n* = 19	*p* Value
ALBUMIN (mg/dL)	4.0 (3.6–4.3)	3.9 (3.8–4.2)	0.92
% HMA	69.9 (68.1–72.6)	68.6 (66.6–72.4)	0.68
% HNA1	27.3 (24.8–29.2)	27.3 (25.5–29.7)	0.92
% HNA2	2.8 (2.6–3.3)	3.2 (2.6–3.6)	0.54

Data are presented as medians (25th–75th percentile). Differences between the groups were assessed by the Mann–Whitney U test.

## Data Availability

The data presented in this study are available from the corresponding author upon reasonable request.
